# Omics Application in Animal Science—A Special Emphasis on Stress Response and Damaging Behaviour in Pigs

**DOI:** 10.3390/genes11080920

**Published:** 2020-08-11

**Authors:** Claudia Kasper, David Ribeiro, André M. de Almeida, Catherine Larzul, Laurence Liaubet, Eduard Murani

**Affiliations:** 1 Swine Research Unit, Agroscope, La Tioleyre 4, 1725 Posieux, Switzerland; 2 Departamento de Ciências e Engenharia de Biosistemas, Instituto Superior d’Agronomia, Universidade de Lisboa, Tapada da Ajuda, 1349-017 Lisboa, Portugal; davidribeiro@isa.ulisboa.pt (D.R.); aalmeida@isa.ulisboa.pt (A.M.d.A.); 3 Génétique Physiologie et Systèmes d’Elevage (GenPhySE), INRA, 24 chemin de Borde-Rouge-Auzeville Tolosane, 31326 Castanet Tolosan, France; catherine.larzul@inrae.fr (C.L.); laurence.liaubet@inrae.fr (L.L.); 4 Leibniz Institute for Farm Animal Biology (FBN), Institute of Genome Biology, Genomics Unit, Wilhelm-Stahl-Allee 2, 18196 Dummerstorf, Germany

**Keywords:** genomics, epigenomics, transcriptomics, proteomics, metabolomics, biomarkers, swine, animal welfare, damaging behaviour

## Abstract

Increasing stress resilience of livestock is important for ethical and profitable meat and dairy production. Susceptibility to stress can entail damaging behaviours, a common problem in pig production. Breeding animals with increased stress resilience is difficult for various reasons. First, studies on neuroendocrine and behavioural stress responses in farm animals are scarce, as it is difficult to record adequate phenotypes under field conditions. Second, damaging behaviours and stress susceptibility are complex traits, and their biology is not yet well understood. Dissecting complex traits into biologically better defined, heritable and easily measurable proxy traits and developing biomarkers will facilitate recording these traits in large numbers. High-throughput molecular technologies (“omics”) study the entirety of molecules and their interactions in a single analysis step. They can help to decipher the contributions of different physiological systems and identify candidate molecules that are representative of different physiological pathways. Here, we provide a general overview of different omics approaches and we give examples of how these techniques could be applied to discover biomarkers. We discuss the genetic dissection of the stress response by different omics techniques and we provide examples and outline potential applications of omics tools to understand and prevent outbreaks of damaging behaviours.

## 1. Introduction

**Figure 1:**
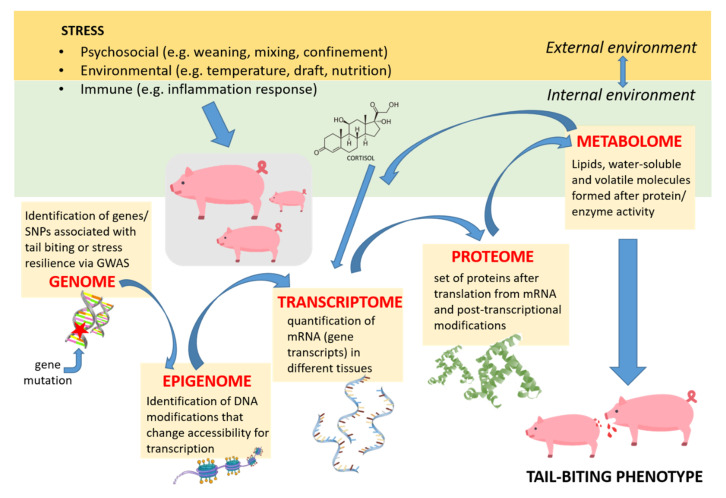
Overview of omics techniques based on a pig model illustrating tail biting. Using genomics, entire genes or single nucleotide polymorphisms (SNPs), which are associated with damaging behaviours or stress susceptibility, can be identified via genotyping or by sequencing the entire genome in a genome-wide association study (GWAS) [17,18]. Transcriptomics allows the quantification of gene expression in relation with the stress response [19]. Proteomics examines the entire set of proteins formed after mRNA translation and subsequent post-translation modifications (PTMs). Particular protein species can be quantified in response to welfare alteration. Metabolomics studies the metabolites (lipids, water soluble and volatile molecules) that are necessary for protein/enzyme activity to occur or are formed because of these reactions [17]. (Figure was created using a pig icon by Freepik from www.flaticon.com).

Increasing robustness, resilience and efficiency of livestock are pivotal topics in animal science because of their importance to profitable meat and dairy production, but also for ethical, animal welfare and sustainability reasons [1,2]. In this review, we investigate and discuss potential benefits of omics technologies for optimizing stress resilience and reducing damaging behaviour in livestock, in particular pigs. Resilience, i.e., the capacity to quickly return to normal functioning after disturbance [3], depends on intrinsic adaptation mechanisms at diverse functional levels, including the genetic and epigenetic make-up, and on their interaction with extrinsic, environmental factors. A better understanding of those factors and how they interact will help to explain individual variation [4,5]. Recent efforts to improve performance of livestock species through intensified selective breeding, including increasing lean meat content, growth and feed efficiency, have been associated with a negative impact on homeostasis through correlated responses to selection [4,6,7]. As a result, disease tolerance [8] and resilience to stressors [9] can be compromised. This may ultimately lead to severe welfare problems, such as behavioural disturbances including tail biting in pigs. It is generally understood that tail biting occurs when the adaptation capacity of an animal is overwhelmed by accumulating risk factors, such as lack of manipulable material and poor health or challenging climate [10]. Because of the multifactorial background it is difficult to identify the critical trigger and predict tail-biting outbreaks [11]. Therefore, it is of paramount importance to identify animal-based indicators of vulnerable state in order to prevent tail biting outbreaks. It is equally important to understand in more detail the biology and genetic architecture of damaging behaviour, and more broadly of resilience-related traits, to be able to predict which animals are more vulnerable to stress and, therefore, at greater risk of developing damaging behaviour [12]. Besides its negative impact on animal welfare, damaging behaviour also has a negative impact on economic and environmental sustainability of animal production. Genetic improvement of resilience and breeding of animals less prone to damaging behaviour promises a more sustainable solution compared with management changes [1,13,14], particularly when looking at the limited success of management interventions to mitigate the problem of tail biting [15,16]. However, due to the episodic nature and dynamics of tail biting, it is difficult to collect direct records of use for breeding [13]. Especially tail-biters are difficult to identify, whereas victims are easily discernible by their wounds. An enormous amount of time for live observations or video analysis is needed, which hinders routine high-throughput phenotyping. Similarly, resilience is not directly measurable, but a concept that requires measuring experienced stress via proxies or biomarkers over time. The development of generally accepted and reliable biomarkers is not yet so advanced that this could be a common strategy. Therefore, breeding would be facilitated by dissecting these complex traits into biologically better defined, heritable and easily measurable proxy traits. This goal calls for the application of holistic omics approaches (hereafter referred to as “omics”), which are high-throughput molecular technologies that study the entirety of molecules and their interactions on various levels of biological organization. They go from the level of the DNA (genomics), to its accessibility for transcription (epigenomics), the abundance of gene transcripts (transcriptomics), proteins (proteomics) and metabolites (metabolomics), or characterizing microbial communities, for instance in the gut (metagenomics), in a single analysis step (Figure 1). Omics are able to enlighten the causal biology from the genome via intermediate molecular traits to a complex, external phenotype, but also provide means for high throughput, deep phenotyping. Such fine-grained information offers further the chance to attempt decoupling correlated traits in breeding programs and avoid antagonistic relationship between welfare and performance [12]. In this review, we elaborate on general characteristics and benefits of different omics approaches (Section 2), provide examples of omics application for discovery of biomarkers of stress exposure and vulnerability (Section 3), and for genetic dissection of stress response (Section 4). Finally, we provide examples and outline potential application of omics tools and future directions to understand and prevent tail biting (Section 5 and Section 6). In our review, we focus particularly on the stress response as a key biological component of resilience and consequently damaging behaviour. The successful application of omics in exploring the neuroendocrine stress response emphasizes the benefit of dissection of complex traits into biologically well-defined traits.

## 2. The Importance of Omics Technologies for Ethical Pig Production 

All omics approaches are fundamentally holistic because they provide information about the set of identified genes present in an individual or characterize the entire microbiome, the entirety of genes transcribed to mRNA or the proteins and metabolites found at a given moment in a particular cell, tissue, organ, fluid, organism or population. Due to their high-throughput nature, these technologies enable the collection of large amounts of data in the form of molecular profiles that provide information about the function of biochemical pathways and thus the functioning of organisms in a certain study. A particularly useful feature of some omics technologies is the ability to characterize and quantify the variation of molecular activity (e.g., transcripts, proteins and metabolites) between individuals, tissues and cells as well as in response to experimental conditions or ecological, behavioural and temporal changes. As some omics technologies can be conducted in a non-destructive and minimally invasive way, they can be used as diagnostic and prognostic tools.

Like most traits of interest in animal science, for instance growth, metabolic and behavioural characteristics, damaging behaviours and stress susceptibility are complex traits. This means that, even though genetic variation underlies trait variation to a certain degree, the genotype–phenotype map is not straightforward. Relevant loci are likely to be high in number and spread across the whole genome, have epistatic relationships with other loci and interact with the environment [20]. The genetic basis of the stress response in pigs will be outlined in more detail in Section 4. Differences in the genetic makeup only partly explain why individuals vary regarding the susceptibility to stress, the propensity to perform damaging behaviours or the failure to resist being bitten by a conspecific. The physical and social environment and genotype-environment interactions (GxE) provide major contributions to individual variation in behaviours and coping styles [21,22]. Early experience and exposure to stressors during developmental stages that are particularly sensitive to environmental challenges can result in permanent modifications of chromatin accessibility [23], leaving a distinctive epigenetic signature that could be exploited in future research on farm animals. The immediate state or environment of an individual can also influence the way genes are transcribed by the action of hormones and neurotransmitters, thereby altering their level of expression and the amount of protein translated (Figure 1). Thus, due to its complexity, the study of stress susceptibility or damaging behaviours requires a systems biology approach. This means that information from different omics techniques, such as gene polymorphisms, quantification of gene transcripts and proteins, metabolites or hormones have to be integrated to obtain a more complete picture of the processes that result in the observed phenomenon. Integrating information across levels of biological organization and environmental conditions is expected to facilitate the understanding of how causative genetic polymorphisms give rise to different phenotypes. For instance, one can study how the combination of genetic polymorphisms, chromatin modifications, hormones and/or the immune system (“intermediate phenotypes”), [24] together result in a tail biting or stress susceptible phenotype. A range of methods exist that facilitate the integration of multi-omics data, such as network or pathway analysis, principal component analysis (or other data reduction methods) and deep learning [25,26,27,28,29,30,31,32]. However, as discussing these methods would go far beyond the scope of this review, we provide a cursory overview of software packages that implement multi-omics integration in Table 1.

## 3. Biomarkers for Stress Resilience in Farm Animals

**Figure 2:**
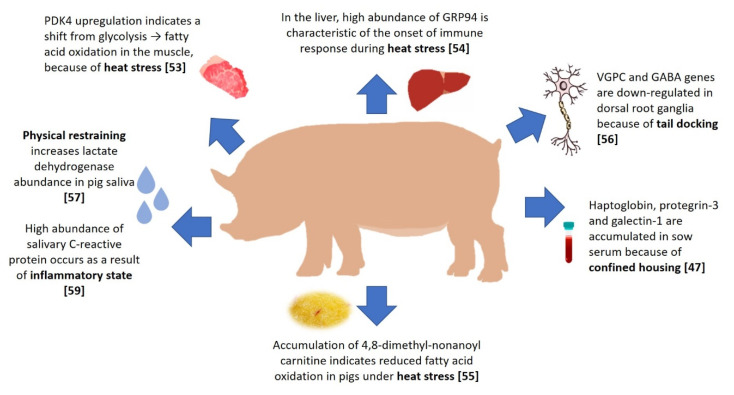
An overview of biomarkers related to welfare issues in pig production.

Several dimensions define stress: nature, intensity, duration and frequency, as well as predictability and controllability. Pigs are confronted with several types of stressors during their lives [45]. Psychosocial stressors occur when piglets are weaned, mixed with unfamiliar conspecifics or during individual confinement of sows [46]. Environmental stressors include climate, noise, ammonia concentrations, suboptimal nutrition and road transport. Immune challenges during disease or after vaccination represent another type of stressors. Several physiological systems, such as the neuro-endocrine hypothalamic pituitary adrenocortical (HPA) axis, the autonomous nervous sympathetic-adrenal-medullary (SAM) system, and the immune system are implicated in the susceptibility to stress [47], whereby feedback regulation represents a crucial protective mechanisms from excessive action of these systems and consequently biological costs (termed allostatic load; [48]). Omics approaches can help to decipher the contributions of the various systems in terms of their (re)activity and kinetics, and identify candidate molecules that are representative of different physiological pathways. Several biomarkers have been suggested as indicators of various traits important for animal production. Studying the proteome associated with pre-slaughter stress in ruminants yielded a range of candidate proteins that underlie the conversion of muscle to dark, firm and dry muscle [49,50,51,52]. Specific findings in pigs, such as on heat stress [53,54,55], tail docking [56], confined housing [47], physical restraining [57], road transport [58] and inflammatory state [59] have potential to be applied to the stress response in pigs (Figure 2). The main neuroendocrine factors that are currently used as biomarkers describing the acute stress response are cortisol and adrenocorticotropic hormone (ACTH) for the HPA axis, and adrenaline, noradrenaline, and chromogranin A for the SAM system, typically measured in blood or saliva [45]. Long-term activity of the HPA axis or exposure to repeated stress can be revealed by measuring the mass and structure of the adrenal gland [60,61]. Behavioural responses, such as changes in coping style [62] and exploratory [63] and sickness behaviour [46], also reflect the amplitude and duration of the stress response and individual propensity to adapt to stress. 

Holistic omics approaches promise to yield novel biomarkers for reactivity and vulnerability to stress, but also provide insight into stress biology. The development of biomarkers will enable fast and reliable tests that complement behavioural observations. The choice of which omics technique to use depends on the physiological level one wants to evaluate and towards what purpose. The discovery and development of heritable biomarkers through genomics may inform future genetic studies (see Section 4 for more details) and improve sire/dam selection programmes (see Section 5 and Section 6 for more details). Furthermore, genomic biomarkers are particularly well suited for the early prediction of general stress susceptibility or the predisposition to tail biting. However, if the objective is to assess the current or recent exposure to stress, using proteomics and metabolomics to search for associated biomarkers might be a preferable approach. Proteomics and metabolomics both integrate signals from genetic and environmental contributions. By presenting a snapshot of proteins and metabolites, they provide information about the functional state of an individual, which is close to functional endpoints and phenotype [64]. More generally, omics technologies have the potential to provide novel insights into stress biology and its links to production traits, health and welfare. Single biomarker molecules or types of biomarkers (e.g., transcripts, proteins/peptides or metabolites) will not capture the complexity of the stress response very well [45,64], and they are often not specific enough to indicate exposure to stress [47]. The combination of complementary omics techniques is, therefore, well suited to find unique signatures of molecular patterns underlying stress susceptibility. In addition to identifying biomarkers, using complementary omics techniques will increase our general biological understanding of the interplay of diverse physiological systems that are implicated in stress susceptibility and stress response in normal functioning and in a disturbed state. The candidate molecules identified in such studies, which have the potential to allow the diagnostics of specific states, such as the vulnerability to fall victim to tail biting or to engage in this damaging behaviour, can then be further tested and validated as biomarkers.

A good biomarker should be assessed from a sample that is easily obtainable and allows minimally invasive collection, for instance from saliva, urine or faeces, hair or feathers, rather than from blood. Ideally, the same individual can be sampled repeatedly [47]. It is of utmost importance that biomarkers enable objective measurement and evaluation [64] and are specific for a particular state or disease [65]. Thus, biomarker molecules should be sensitive and statistically clearly and consistently associated with a specific normal or pathogenic process [47,64,65] or the risk of an individual to develop a disease [65]. Measurement of biomarker molecules should be simple and cost-effective, and the target molecule should be stable [47,65]. Assessment of biomarkers early in life allows early detection and intervention [64] if they are predictive of future outcomes (see Section 6). To validate biomarkers, the application of suitable statistical approaches [65] as well as an established scientific framework and a substantial body of evidence are crucial [64]. Each omics technique provides complementary information and has the potential to identify different potential candidate biomarker molecules (Table 2). Heritable biomarkers of vulnerability and predisposition can be identified using genomics (see Section 4). Environmental challenges may be revealed by epigenetic biomarkers, and include histone modifications or DNA methylation patterns. A landmark in the research and diagnosis of long-term impact of adverse life events has been the discovery of epigenetic programming of stress responsiveness in the offspring by maternal care after birth in rodents [66], changing hippocampal expression [67] and leaving a striking epigenetic signature in and around the glucocorticoid receptor locus [68]. Similar epigenetic signatures have been discovered in humans with a history of childhood abuse [68]. Epigenetic biomarkers have been developed for diagnosis of metabolic diseases and, perhaps of most interest here, neuropsychiatric disorders in humans [69,70,71]. In pigs, the influence of early-life stress has been extensively studied and documented (reviewed in [72]; however, with few exceptions [73], the epigenome alterations remain largely unexplored. Transcriptomics can be used to associate gene-expression profiles or profiles of non-coding RNA (for instance, long non-coding RNAs, lncRNAs; micro-RNAs, miRNAs; and small interference RNAs, siRNAs) with stress vulnerability or resilience phenotypes [74,75]. MiRNAs seem to be especially promising for biomarker development because miRNAs circulating in the blood have been shown to reflect resilience or vulnerability to social stress in rats [74]. Salivary miRNAs that reflect a particular condition well have even greater prospects of application in animal welfare due to the reduced invasiveness of saliva compared to blood samples. Following tail-docking without anti-inflammatory treatment, the concentrations of three salivary miRNAs were increased compared with a group that received treatment [76]. Interestingly, the combination of just two miRNA concentrations, miR-19b and miR-365, was sufficient to distinguish treatment groups [76]. This type of molecule harbours potential for translational research since they are highly conserved across species [77]. However, single miRNAs might not be specific to a particular disorder [75]. Proteomics is well suited to screen biofluids, such as blood, urine or saliva, for specific proteins associated with a specific condition or state [65], and in particular, to assess stress in farm animals [47]. The metabolome is informative about the functional state of an organism, and therefore, close to functional endpoints and phenotypes [64]. This aspect and the fact that the metabolites are often not breed- or species-specific [64] suggest a high potential of metabolomics particularly for translational studies. Due to the limited specificity of individual biomarkers, panels of biomarkers may yield better outcomes [47,64].

## 4. Genetics of Stress Response

### 4.1. Quantitative and Molecular Genetics

Genetic variants are the ultimate biomarkers when it comes to prediction of predisposition or potential to express a specific trait. The genetic material of an individual. i.e. DNA, is available early in development, and is easily obtained from any cellular material available, allowing early prediction of its genetic potential. Currently single nucleotide polymorphisms (SNP) are the genetic variants of choice for genetic studies due to their high abundance (several millions or one SNP per <1kbp segregating in a population), stability of the matrix (DNA), mutational stability (i.e. consistency), and categorical nature (most SNPs are biallelic [83]). These features make SNPs perfectly suitable for high throughput interrogation required for genome-wide analyses. 

So far, genetic studies of neuroendocrine and behavioural stress responses in farm animals including pigs are scarce due to difficulties to record adequate phenotypes, particularly under practical conditions (see Introduction). Therefore, most genetic studies were carried out under experimental conditions and focused particularly on HPA axis activity, or more specifically on cortisol production and related pathways. The contribution of genetic factors to HPA axis activity is now well established, demonstrated for example, by distinct breed differences. Domestic pigs show less adrenocortical activity than wild boars [84], which is in line with insights gained from domestication experiments in several model species based on selection for tameness [85,86,87,88]. Cortisol is a pleiotropic hormone influencing almost all physiological systems including the immune system and energy homeostasis [89]. Therefore, apart from a domestication process based on selection on tameness/docility, differences in HPA axis activity and cortisol production were likely driven further by breeding for greater lean deposition as indicated for example by the observation that breeds showing greater adiposity, e.g., Chinese breeds, typically feature greater cortisol concentrations compared with elite commercial breeds [84,90,91]. However, while cortisol production is linked with a wide range of production traits (i.e., feed efficiency [92], piglet survival [93,94]), this relationship is quite complex and often ambiguous [95,96]. This exemplifies the limited explanatory power of individual endocrine biomarkers and hence requirement for a holistic approach integrating other parts of the signalling cascade. 

For better understanding of the genetic determinism of HPA axis activity, several studies focused on cortisol concentration, at rest or under challenge. Kadarmideen and Janss [97] estimated a heritability value of 0.40 for basal cortisol concentration measured in urine, but the heritability values reached 0.7 when cortisol concentration was adjusted to creatinine concentration, which allowed for urine dilution by water intake to be taken into account. Larzul et al. [98] estimated a heritability value of 0.19 for basal cortisol concentration in plasma at 6 weeks of age. The cortisol concentration in plasma measured one hour after an injection of ACTH had a heritability value of 0.68. The genetic correlation between the two measures was 0.94 [98]. Consistent with the considerable heritability, genome-wide linkage or association analyses evidenced several trait-associated loci (QTL) for HPA axis activity or SAM responses (a comprehensive database of QTLs mapped in farm animal species, Animal QTLdb, can be accessed at https://www.animalgenome.org/). Two main QTL regions include the distal end of chromosome 7, harbouring the *SERPINA6* locus coding for corticosteroid binding globulin (CBG) [61,99,100,101] and the distal end of chromosome 2, harboring the *NR3C1* locus coding for glucocorticoid receptor (GR) [61,102,103]. 

Beside HPA axis function, coping strategy also shows a considerable genetic basis. This is typically tested using a so-called backtest [104]. In the test, pigs are placed in a supine position, and latency, duration, and number of struggling bouts is used to classify the coping style [105]. Heritability estimates for performance in the backtest range between 0.10 and 0.50 [105,106,107,108]. The main QTL for backtest behaviour was detected on chromosome 12 [109]. Importantly, an association of two peak SNPs in this QTL, particularly rs10720116 (ASGA0055092) located in a prominent functional-positional candidate gene *PER1*, could be independently validated in a crossbred population. Only few minor QTLs were detected for behavioural stress responses [99], which is consistent with the expected highly complex background of such traits. Individual differences in stress responsiveness may also be described indirectly, by exploiting routinely measured traits influenced by stress such as feed intake or growth [110]. Cross et al. [111] analysed the impact of heat stress, measured by temperature humidity index (THI, categorized as normal, alert, danger, emergency), on feed intake in commercial crossbred pigs. Interestingly, breed-associated differences were observed, with Landrace-sired pigs being more heat stress susceptible compared with Duroc or Yorkshire sired pigs. Highest heritability (0.21) showed a change in feeding behaviour between normal and danger THI. QTL on chromosomes 1, 7, 14 and 17 showed the most pronounced effects on changes in feeding pattern due to different THI. Functional annotation of genes in the QTL regions revealed pathways associated with the immune system as overrepresented. The QTL regions contained several heat shock proteins, such as *DNAJA4*, *HPS90AA1* and *HSP90AB1*, which are obvious candidates due to their role in protecting protein function during heat stress.

Whereas several QTL for different stress-response related traits were mapped, including robust signals such as the QTL for cortisol on SSC7, identification of the causal genetic variants, termed quantitative trait nucleotides (QTN), has proven difficult. So far only one causal QTN has been identified, a missense SNP c.1829C>T (rs335303636) in exon 6 of *NR3C1* with major effect on HPA axis activity [61]. The resulting amino acid exchange Ala610Val in the ligand binding domain (LBD) leads to a gain-of-function, enhancing steroid sensitivity of GR presumably by changing the structure of the LBD and strengthening the LBD/ligand interaction [112]. The QTN segregates in diverse commercial pig breeds (German Landrace, German Large White, Pietrain, Duroc) and shows consistent association with cortisol production [61]. Because GR is involved in negative feedback regulation of the HPA axis activity, the enhanced receptor sensitivity (i.e., hypersensitivity) likely leads to compensatory downregulation of the HPA axis early in ontogeny [113]. While the effect on adrenal function is most pronounced, the mutation influences also activity of the central branch of the HPA axis, including corticotropin releasing hormone (*CRH*) and vasopressin expression [113]. Since these latter neuropeptides are involved also in the regulation of behaviour and SAM, the mutation might have a broader impact on stress responses. In contrast, although *SERPINA6* provides a plausible candidate causal locus for the QTL on chromosome 7 [114], the causal genetic variant remains elusive. Nucleotide diversity of *SERPINA6* is quite high [115]. Guyonnet-Duperat et al. [116] characterized four amino acid substitutions segregating in the mapping population (Meishan×Large White). They found that the substitution Gly307Arg, originating from domestic pigs [115], increases CBG capacity and decreases CBG affinity for cortisol. However, the amino acid substitutions do not fully explain the QTL in any of the populations where the QTL has been detected [114,116,117]. Apart from coding variation, *SERPINA6* function is apparently diversified by regulatory variation [114,117]. Thus, the causal effect might result from an interaction of the regulatory with the coding variation. 

It is interesting to note that the main loci, *NR3C1* (GR) and *SERPINA6* (CBG), affecting the HPA axis in pigs are not those directly involved in the production of HPA axis hormones but rather genes involved in regulation of its activity or signal transduction. This may indicate greater evolutionary or functional constraint on genes involved in production of HPA axis hormones, or *vice versa*, that variation in regulation might be better tolerated or even beneficial due to reduced pleiotropy and greater flexibility.

### 4.2. Functional Genomics

**Figure 3:**
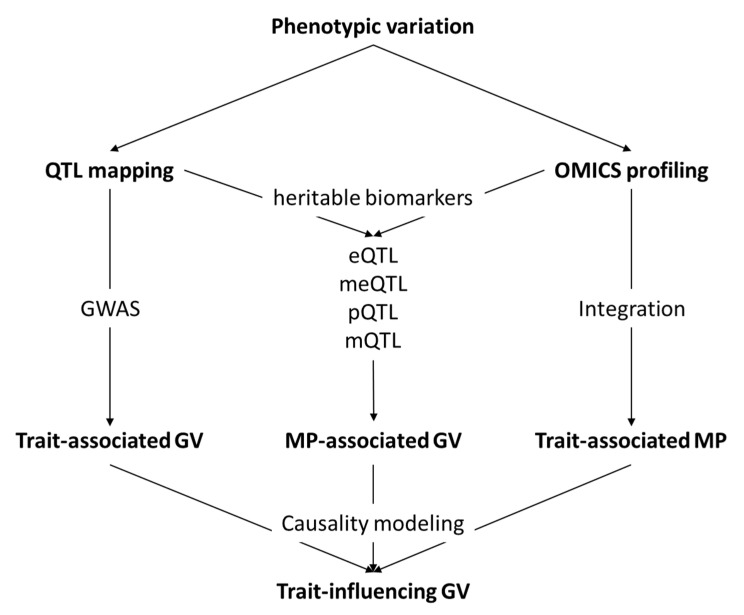
General outline of the integration of omics and genetic analyses in the dissection of the genetic background of complex traits. GV: genetic variation, MP: molecular pattern, QTL: quantitative trait locus, GWAS: genome-wide association study, eQTL: QTL for gene expression, meQTL: QTL for DNA-methylation, pQTL: QTL for protein expression, mQTL: QTL for metabolite, WGCNA: weighted gene co-expression network analysis.

Many obstacles hamper genetic dissection of complex traits such as stress resilience and damaging behaviour. First, QTL detection requires collection of suitable phenotypes in a large cohort of informative animals, which is, as mentioned in previous sections, difficult for stress resilience and damaging behaviour. Here, omics approaches might provide easily measurable biomarkers, which can be measured in large cohorts with high throughput. Genome-wide association studies are per se hypothesis-free and can be performed for any trait or parameter with reasonable heritability. Another obstacle is the biological and genetic architecture of the trait of interest. Complex phenotypes, as typified by the multifactorial damaging behaviour, can be dissected into biologically better-characterized and genetically funded components or intermediate traits by applying omics approaches. This dissection greatly facilitates QTL detection (Figure 3) and may provide the basis for selective breeding. In this regard, different omics levels connecting genotype to phenotype have different degrees of genetic foundation, with the transcriptome having the closest functional connection to the (epi)genome. The most powerful studies are those integrating external and intermediate molecular phenotypes at multiple omics level into a systems genetic or genomic approach [25,26]. Furthermore, capturing all genetic variants, including all causal nucleotides, by whole genome sequencing (WGS) will further increase power and precision of QTL mapping [118], but this is methodically challenging. Finally, the most challenging step proved the dissection of the molecular background of identified QTLs. Besides high sequence variation and allelic heterogeneity as in the case of the *SERPINA6* locus, another obstacle is linkage disequilibrium, which makes it difficult to distinguish between causal and linked variants. Here, it is essential to complement genetics by functional approaches, including omics. While in most cases, major QTLs are caused by coding variants [119], such as GRAla610Val; it is generally assumed that regulatory variation is the common cause of variation in complex traits [120,121]. For a large proportion of genes, their expression is heritable and a considerable part of their variation can be explained by genetics [122]. Genome-wide mapping of expression QTL (eQTL) [123], also referred to as “genetical genomics” [124], has thus become a popular approach to dissect complex trait genetics. Genes whose eQTL are mapped are typically selected based on either positional candidacy and/or correlated expression to variation in the trait of interest. Ponsuksili et al. [103] used this approach to identify genes and networks in skeletal muscle and liver either responding (downstream) to, or influencing (upstream) circulating cortisol in the pigs. First, they identified genes whose expression is significantly correlated to plasma cortisol concentration. Subsequently, they applied GWAS to find eQTL for correlated genes and QTL for plasma cortisol concentration. Finally, the results were integrated using network edge orienting (NEO), a causal modelling algorithm [39]. The study yielded two genes in the muscle and 26 genes in the liver potentially influencing cortisol production, along with 25 and 70 responsive genes, respectively. This study illustrates a suitable approach to resolve causality of relationships between gene expression and manifestation of a trait of interest. Ponsuksili et al. [109] employed eQTL mapping also to resolve the QTL on chromosome 12 for coping behaviour. For this, expression of 37 positional candidates in the hypothalamus was measured. For eight genes, *cis*-eQTL (i.e., proximal to the gene itself) were mapped. The prospective candidate turned out to be *CTC1*, featuring overlapping SNPs for the coping behaviour QTL and *cis*-eQTL. However, the causal variants still await discovery. Beside protein-coding genes, also expression of non-coding RNA is influenced by genetics [125]. However, studies exploring stress-response-related non-coding RNA profiles in a genetic context are scarce (but see [78,79]).

Pinpointing the causal variant is particularly difficult for regulatory variants, as shown, for example, for the porcine *ADRB2* [126,127], a receptor involved in SAM system signalling. Expression of *ADRB2* is diversified by several haplotypes depending on tissue context. Furthermore, gene regulation is by far less well understood compared with genetic code and regulatory elements in farm animal genomes are so far poorly annotated. Therefore, the international FAANG (Functional Annotation of Animal Genomes) Consortium was initiated [128]. This uses different functional genomics approaches to generate a high-quality, and high-resolution reference map of functional genome elements in different farm animal species, including pigs. The functional genome annotation will include a comprehensive catalogue of transcribed loci (coding and noncoding), chart of the chromatin landscape (histone modifications, chromatin accessibility and spatial conformation) showing regulatory elements and their interactions, and a chart of the epigenomic landscape represented by DNA-methylation. Such functional genome map will improve our understanding on how environmental factors shape the epigenomic landscape, gene activity and ultimately phenotypic variation, and further greatly facilitate identification of potentially functional, and consequently potentially causal, genetic variants. With increasing number of identified causal variants, knowledge about molecular mechanism of their action and genetic mechanisms driving their occurrence and divergence will also accumulate and accelerate identification of further loci. First, sequence based methods for the evaluation of functional consequences and prioritization of genetic variants such as pCADD [129], were developed for farm animals, and it can be expected that these will be further expanded using data generated by FAANG. Furthermore, first methods incorporating functional genome information into genetic studies and genomic prediction are emerging [130,131].

An alternative approach to eQTL mapping for the identification of regulatory polymorphisms is analysis of allele-specific expression or allelic expression imbalance [132]. However, in the context of stress and behaviour-related genes, this sensitive approach has, so far, only been applied to individual candidates in pigs, such as *ADRB2* mentioned above [126]. Maroilley et al. [133] applied this approach in combination with eQTL mapping to analyse the genetic architecture of variation in gene expression in the blood (peripheral blood mononuclear cell, PBMC) in pigs. Eleven percent of genes expressed in PBMC showed evidence for allelic expression imbalance, i.e., for *cis*-regulatory variation, indicating considerable contribution of genetics to gene expression variation as also reported, for example, for mice [134].

## 5. Omics to Study Tail Biting

Tail biting is a damaging behaviour regularly observed in group-housed pigs [135] and it is the source of considerable health problems in pig breeding. Tail docking a few days after birth is currently the main procedure to prevent tail biting, but since 1994, routine tail docking has been forbidden in many European countries by the European legislation for welfare reasons [136]. Genes involved in neuropathic and inflammatory pain pathways were shown to be differentially expressed between tail-docked and sham-treated pigs until 16 weeks after amputation [56], indicating a long-term disturbance of well-being. Tail biting is a multifactorial problem and its occurrence is, therefore, difficult to predict. It is considered as a consequence of living in a stimulus-deprived environment in combination with the high density of animals per pen. Tail biting reflects the primary need to forage and explore the environment. Nevertheless, it is considered a sign of stress related to many risk factors, such as the absence of enrichment, comfort (temperature, humidity, air quality, and light) and cleanliness, good health (for instance, gastrointestinal comfort), competition for food, sufficient quality and quantity of food and water, age and genetic predisposition [137]. Genetic differences are evident in that breeds differ in their tendency to become victims of tail biting, e.g., Yorkshire pigs are bitten more often than Landrace pigs [138], and to bite objects (oral rope manipulation) and conspecifics (the latter is shown more often by Duroc pigs than by Large White and Landrace pigs [139]). However, tail biting has a low heritability around 5% [7]. When the genotypes of pen mates are included in the analysis (indirect genetic effects), heritability can increase up to 24% (L. van der Zande, unpublished). Indirect genetic effects can contribute to the heritable variation of a trait, and therefore, increase heritability. Moreover, selection for leaner meat and carcasses is genetically correlated with tail biting [7,135,140]. Careful evaluation of the effects of breeding programs and selection experiments on the propensity for tail biting is, therefore, of vital importance. However, tail-biting behaviour is relatively difficult to examine, e.g., injuries to the carcass were frequently observed, which allows the identification of the victims but not the biter [141]. Tail biting is a relevant trait for pig breeders because it impairs animal welfare and causes economic losses to the farmer. Its low heritability in combination with difficulties in obtaining reliable phenotypes and its complex genetic architecture, including also indirect genetic effects (i.e., gene–gene interactions in different individuals) make it particularly difficult to include it in a breeding program. Omics approaches are not yet widely used in breeding nuclei due to the methodological challenges they pose to be routinely implemented in field conditions. Sample collection and phenotyping have to be streamlined to allow integration of collection into routine procedures. The development of easy-to-use and robust collection methods is necessary to guarantee stable outcomes under conditions that are not fully controllable. 

Owing to these difficulties, only a few studies using omics technologies have been published to date on tail biting behaviour. Genetic association studies have identified a QTL for the number of lesions in Yorkshire pigs after mixing on chromosome 11 [142]. Being a victim of tail biting was associated with genomic regions on chromosomes 1, 9, 18 and an unassigned region [143]. One association with being a tail-biter could not be assigned to a genomic region and another one was found on chromosome 16 [143]. Thus, it seems that the two traits (being a victim and being a tail-biter) are not genetically linked. The combination of the low availability of genetic loci studies for tail-biting behaviour, the difficulty of distinguishing the initial biter in a group of pigs and the fact that relatively few QTLs and no biomarkers are known, calls for more holistic studies to better understand the architecture of this undesirable trait. In addition to genetics, information on different levels of post-genomic regulation needs to be explored, which can be obtained through transcriptomic, proteomic, metabolomics and even metagenomics studies. While there are few genetic studies of damaging behaviour in pigs or chickens, research on neuropsychological disorders in humans could provide a way forward. A gene expression study in blood allowed the identification of a set of six genes necessary to discriminate obsessive–compulsive disorder from other psychiatric disorders (schizophrenia or depression) and from controls with an accuracy of 86% [144]. In order to gain power to identify candidate genes, GWAS and transcriptomic data obtained in humans and mice have been combined to study aggressive behaviour [145]. Significantly enriched biological pathways across species were supplemented with phenotypic information to define a ranked list of genes involved [145]. *RBFOX1* (RNA Binding Fox-1 Homolog 1), one of the top genes in the lists of significantly enriched signalling pathways in different species, is a regulator of neuronal development [146]. This gene was found to be associated with aggressive behaviour in Drosophila, mice and dogs and in depression, the measurement of well-being, cognitive functions and conduct disorder in humans (reviewed in [147]). Fernàndez-Castillo et al (2020) [147] confirmed the added value of using data from different omics together with other phenotypic studies (e.g., imaging, transgenic mice), even if from different species, to propose more reliable biomarkers and candidate genes. 

Gene expression studies in the brains of victims, performers and “neutral” pigs help shed light on the genes and physiological pathways that have a role in tail biting. Brunberg and colleagues described the transcriptomic analyses of brain structures (hypothalamus and prefrontral cortex) *post-mortem* from pigs classified as tail biters, victims or neutral pigs [141,148,149], and whether they were able to resist tail biting [148]. More genes were differentially expressed between neutral pigs vs biters and victims than between biters and victims in the hypothalamus and in the prefrontal cortex [149]. Identifying neutral behaviour, and in particular, resistance to damaging behaviour could thus be an interesting starting point for further genetic studies. Some of the genes found differentially expressed were related to production traits in pigs (for instance, *PDK4*—pyruvate dehydrogenase kinase, isozyme 4; [150] or related to hypersociability in mice (e.g., *GTF2I*—general transcription factor II-I gene; [151]) The *PDK4* gene has also been identified as a possible biomarker of heat stress [152]. Another important gene, *EGF* (epidermal growth factor), was more highly expressed in biters/victims than in neutral pigs [149]. A polymorphism in this gene has been implicated in novelty seeking [153] in a Finnish human population. Two other genes with potential implications for production traits in pigs were over-expressed in neutral pigs [148], the *GHRL* gene coding for ghrelin, which is a hormone regulating eating behaviour, and the *COMP* (Cartilage Oligomeric Matrix Protein) gene [149]. The latter was previously identified as being involved in feather pecking in hens [154]. The functions of the identified genes all correspond to production traits (growth, feed intake, leaner carcass) and to behavioural traits. This is coherent with the idea suggesting that genetic selection for production traits may have also selected damaging behaviour as an undesirable side effect. 

The dopaminergic and the serotonergic systems might be highly relevant for the development of biomarkers for tail biting in pigs. The serotoninergic system [155] is involved in many physiological functions as temperature, appetite, sleep, pain, motricity and in cognition (emotion, memory, attention, decision making). The dopaminergic system has an important role in the reward system, influences exploration behaviour and emotional reactions such as fear and anxiety. It is important for coping with stressful environmental conditions and frustrations [156,157]. In pigs, a link has been observed between stress or fear and the serotoninergic system. Tail biters and victims were found to differ in their whole blood and blood platelet concentrations of serotonin (5-HT) from non-tail biters or non-victims [158]. While both tail biters and victims have less whole blood and blood platelet 5-HT than non-biters and non-victims, only biters also had a greater blood platelet uptake velocity [158]. The assessment of peripheral serotonin concentrations may therefore not be informative enough. Peripheral blood serotonin was decreased in biters in comparison to victims [158], similar to what was observed in humans with mental disorders and in laying hens performing feather pecking. Greater concentrations of serotonin were observed in the prefrontal brain of tail-biters compared with victims [159]. Serotonin is involved in the expression of feather pecking behaviour in laying hens, a similar behaviour to tail biting, (reviewed by [160]). Hen lines with high feather pecking tendency have a decreased serotonin content in the brain at young ages, but greater content as adults [160]. The dopaminergic system appears to have a role in feather pecking in hens, since the administration of an antagonist of the dopaminergic system, haloperidol, reduced the occurrence of this damaging behaviour [161]. Feather pecking has been associated with two neighbouring genes involved in dopamine and serotine pathways, one coding for the dopamine D4 receptor (*DRD4*) and one for the deformed epidermal autoregulatory factor 1 (*DEAF1*), a repressor of the serotonin receptor transcription [162]. Serotonin and dopamine link damaging and feeding behaviours [160] when nutritional context is also considered as a risk factor to express damaging behaviour. Palander et al [163] have shown that in pigs, victims of tail biting had less plasmatic non-essential amino acids and inorganic phosphate than biters. Those reduced concentrations of nutrients could be related to a reduced ability of intestinal absorption, as indicated by a comparison of the histology of jejunal villi in pens with tail biting compared with pens without damaging behaviour [154]. Nutritional deficiency, compromised gastrointestinal health and associated discomfort, could lead to changes in the gut–brain axis (perhaps including also microbiota) and consequently, to unfavourable behaviour [135,141], pointing to a new and exciting target for omics studies.

Taken together, these studies propose that, despite the difficulties of studying damaging behavioural traits, promising strategies for elucidating this complexity have been suggested and could be applied in pigs. The combination of functional and genome-wide association studies could make it possible to better characterize the trait of interest, identify genes and even polymorphisms that could be used in genetic selection. Ideally, the combination is realized within the same study. However, experimental difficulties could be overcome by using available functional (omics) information coming from different projects or even from other species (reviewed in [145]).

## 6. Conclusions

Even though omics technologies are already widely used in animal science, for instance to assess production traits and meat quality, they have not yet been fully exploited for ethical pig production. Tail biting is a complex trait with substantial contribution from the environment and a complicated genetic architecture (potentially including indirect genetic effects). A genetic contribution to the tail-biting phenotype has been demonstrated, but few studies have found regions associated with the stress response in pigs, often without identifying causal variants. The involvement of variation in regulatory regions is expected, which is best captured with a combination of different omics methods. In particular, the integration of several data sets measured on the same individuals and obtained with different omics platforms promises to unravel the interaction between different levels of biological organization. Omics approaches have potential to aid with the development of biomarkers that can be used as diagnostics and prognostic tools of damaging behaviours. Such biomarkers can be useful beyond damaging behaviours and beyond the species level, and might be used as general proxies of welfare in different contexts and for genetic selection of stress-resilient pigs. To achieve this goal, the development of automated, low-input but high-throughput phenotyping strategies that can be easily implemented under field conditions is necessary. Equally important is an improved understanding of the biology of the stress response and the molecular pathways that are implicated in the stress response in different growth stages and environments. Phenomena such as tail biting could be thus dissected into biologically simpler intermediate traits, which have a clearer genetic basis. Ongoing large-scale efforts for improved and more meaningful gene annotation of farm animals will certainly help. Apart from the development of biomarkers, which is a long-term effort, methodological improvements in phenotyping and data integration will facilitate the adoption of omics techniques to study tail biting at the nucleus farm level in the near future.

## Figures and Tables

**Table 1:** Overview of some free bioinformatics software for the integration of information across several omics techniques.

Name	Characteristics	Integration of Types of Omics	Type of Analysis	Reference and URL
**Cytoscape**	standalone software	Mainly protein–protein, protein–DNA and DNA–DNA interactions, but plug-ins (apps) are available for all types of omics	Provides tools to visualize complex molecular and genetic interaction networks, but also network analysis, enrichment analysis, ontology analysis and pathway analysis (e.g., KEGG) is possible.	[33] https://cytoscape.org/
**MOFA**	R package (via Bioconductor)	All types (multi-omics)	**M**ulti-**O**mics **F**actor **A**nalysis enables the unsupervised integration of heterogeneous data sets via a generalization of principal components analysis. MOFA implements hidden factors of biological and technical sources of variability and represents integrated data in an interpretable low-dimensional form.	[34] https://github.com/bioFAM/MOFA
**LUCID**	R package (via CRAN)	Mainly genomics and metabolomics; integration of phenotypic data	Uses **L**atent **U**nknown **C**lusters with **I**ntegrated **D**ata models to distinguish unique genomic, exposure and informative biomarkers or omics effects. Latent underlying relationships with phenotypic traits are estimated in cluster estimations using directed acyclic graphs (DAG). Prediction of phenotypes possible. Visualization of data integration with Sankey diagrams.	[35] https://github.com/USCbiostats/LUCIDus
**MultiDataSet**	R package (via Bioconductor)	Epigenomics, transcriptomics; assay data, feature data, phenotypic data stored in single object	Does not provide tools for analysis itself, but constructs an R data-storage object that contains multiple data sets, making managing and subsetting multiple and non-complete data sets possible. This data set can be plugged in to other R packages for analysis, for instance for multivariate co-inertia analysis (MCIA in *omicade4*) or clustering of multiple tables (in *iClusterPlus*)	[36] https://bioconductor.org/packages/release/bioc/html/MultiDataSet.html
**Logicome Profiler**	standalone Unix software	Applied to genomics and metagenomics, but applicable to any omics data	Detects statistically significant triplet logic relationships from a binary matrix dataset (indicating connection, for instance co-occurrence, co-expression). Applies Logic Analysis of Phylogenetic Profiles (LAPP) method, which is based on normalized mutual information, to phylogenetic profiling data, but also applicable to gene co-expression and pathway data.	[37] https://github.com/fukunagatsu/LogicomeProfiler
**CoCoNet**	R package (via Github)	Integration of GWAS and gene-expression data	**CO**mposite likelihood-based **CO**variance regression **NET**work model to identify trait-relevant tissues or cell types. Uses covariance regression network models to express gene-level effect measurements for a given GWAS trait as a function of the tissue-specific co-expression adjacency matrix.	[38] http://www.xzlab.org/software.html
**NEO**	R package (via CRAN)	Integration of GWAS and gene-expression data	**N**etwork **E**dge **O**rienting infers directed gene networks by integrating gene-expression data with genetic marker data and compares them with structural equation models	[39] https://horvath.genetics.ucla.edu/html/aten/NEO/
**WGCNA**	R package (via CRAN)	Mainly gene-expression data, but can be applied to other omics	**W**eighted **G**ene **C**o-expression **N**etwork **A**nalysis is used to find clusters, relating modules to one another and to external sample traits and calculates module membership measures. This approach facilitates gene screening and the identification of biomarkers.	[40,41] https://horvath.genetics.ucla.edu/html/CoexpressionNetwork/Rpackages/WGCNA/
**DIABLO in mixOmics**	R package (via Bioconductor)	All types (multi-omics)	Multivariate methods to analyse and visualize high-dimensional datasets (number of variables larger than number of samples). Complementary information from several data sets measured on the same N individuals, but across multiple omics data sets is combined to gain a better understanding of the interplay between the different levels of data that are measured (‘N-integration’). Data dimensions are reduced by applying sparse generalized canonical correlation analysis (SGCCA).	[42,43,44] http://mixomics.org/mixdiablo

**Table 2:** Putative biomarkers for experienced stress or stress susceptibility in livestock obtained by different omics approaches.

Omics Type	Molecule Type	Molecule Name	Biofluid/Tissue	Description	Reference
**epigenomics**	DNA methylation	BCL-2 and RORA PPIEL	postmortem brains and peripheral blood cells	hypermethylation of BCL-2 and RORA genes in patients with autism; hypomethylation of PPIEL in bipolar disorder; hypermethylation of genes involved in brain development and tryptophan metabolism	[78]
	DNA methylation	HTR1A, S-COMT, BDNF1 HTR1E, COMTD1 and MB-COMT	peripheral blood cells	peripheral epigenetic biomarkers of schizophrenia; hypermethylation of HTR1A, S-COMT, BDNF 1 hypomethylation of HTR1E, COMTD1 and MB-COMT	[79]
	DNA methylation	VWF and LRRC32	hippocampus	reduced cognition in pigs in response to early life environmental insults (infection with porcine reproductive and respiratory syndrome virus) is associated with differential methylation and differential gene expression. VWF and LRRC32 are implicated in blood brain barrier permeability and regulatory T-cell activation, respectively.	[73]
**transcriptomics**	miRNA	miR-24-2-5p, miR-27a-3p, miR-30e-5p, miR-3590-3p, miR-362-3p, and miR-532-5p	blood	pre-challenge circulating miRNAs reflect resilience or vulnerability to chronic social defeat in rats	[74]
	miRNA	mir-132	diverse tissues and fluids	associated with post-traumatic stress disorder in humans and animal models in a systematic review; lack of specificity	[75]
	miRNA	miR-19b, miR-27b, and miR-365	saliva	concentrations greater in pigs that received no anti-inflammatory treatment after tail docking than in pigs that received treatment	[76]
	miRNA	range of circulating extra-cellular miRNAs	plasma	after feed deprivation in chicken lines selected for high and low residual feed intake, 23 and 19 miRNAs were found to be differentially expressed between feeding conditions and lines (indicating influence of genetic background), respectively.	[80]
	miRNA	range of circulating extra-cellular miRNAs	plasma	miRNA profiles were different between age classes (26 miRNAs) and lines (5 miRNAs) in dairy cattle. Three miRNAs negatively associated with telomere length, but positively with milk fat yield, mastitis and lameness.	[81]
	mRNA	profile	dorsal root ganglia	3000 genes were differentially regulated between docked and undocked pigs	[56]
	mRNA	Pyruvate dehydrogenase (*PDK4*), heat shock (e.g. *HSPB1*) and oxidative (e.g. *COX1*) genes	*longissimus dorsi* muscle	up-regulated in the muscle of pigs under heat stress, reflecting the transition from glycolysis to fatty acid oxidation during chronic exposure to HS	[53]
	mRNA	profile	liver	A list of genes dose-dependently regulated by glucocorticoids as biomarkers of stress action	[82]
**proteomics**	APP	Pig Major Acute Phase protein	serum	7-fold increase in pigs after road transport	[58]
	protein	*GRP94*	liver	of critical importance at the onset of innate immune response, in pigs under HS. induces an inflammatory response, causing hepatocytes to synthesise haptoglobin (HP) and α-1-antichymotrypsin 2 precursor (*SERPINA3*) to maintain cell integrity.	[54]
	protein	lactate dehydrogenase (LDH)	saliva	significantly increased in the saliva of pigs restrained with a nose snare and in pigs with lameness. (LDH follows adrenaline production)	[57]
	protein	haptoglobin protegrin-3 and galectin-1 β-actin	blood serum	transition of sows from group to individual confined housing caused increase; indicates activation of immune defence and cell damage; indicates synthesis of stress-response hormones	[47]
**metabolomics**	metabolite	4,8-dimetil-nonanoyl carnitine	mesenteric adipose tissue	accumulation of 4,8-dimetil-nonanoyl carnitine, an intermediary of fatty acid oxidation, in this tissue of heat stressed pigs	[55]
